# Self-Directed Learning versus Problem-Based Learning in Korean Nurse Education: A Quasi-Experimental Study

**DOI:** 10.3390/healthcare9121763

**Published:** 2021-12-20

**Authors:** Jaehee Jeon, Sihyun Park

**Affiliations:** 1 Department of Nursing, Gangneung-Wonju National University, 7 Jukheon-gil, Gangneung-si 26403, Gangwon-do, Korea; anesjjh@naver.com; 2 Department of Nursing, Chung-Ang University, 84 Heukseok-ro, Dongjak-gu, Seoul 06974, Korea

**Keywords:** education, nursing students, problem-based learning, self-directed learning

## Abstract

Effective teaching methods are vital for cultivating advanced professional skills in nurses and equipping them with the necessary training. Problem-based learning (PBL) and self-directed learning (SDL) have been consistently used in nurse education. Therefore, their effects on nursing students’ academic performance warrant comparison. This study compared the effects of PBL and SDL on an adult nursing university curriculum. Participants in this quasi-experimental study with a pre-post non-equivalent control group design were 106 third-year nursing students divided into the PBL and SDL groups. Data collection, conducted from April to June 2019, included a pre-test before an eight-week intervention, followed by a post-test. Changes in the scores of each group were analyzed for learning motivation, self-directed learning ability, self-efficacy, learning confidence, learning satisfaction, and academic performance using paired and independent t-tests. The PBL group scored higher on learning motivation, self-directed learning ability, and academic performance than the SDL group. Based on these results, the PBL method was more effective than the SDL method in an adult nursing curriculum. To maximize the learning effect in adult nursing education, it is necessary to apply SDL education, including the PBL method, with a clearer learning process.

## 1. Introduction

Today’s medical environment requires nurses to have clinical reasoning abilities, collaboration skills, and the ability to identify and solve patient problems. The foundation of these skills includes the capacity to accurately and comprehensively understand basic medicine and professional nursing, and appropriately apply this knowledge in clinical settings [1]. To ensure that students acquire these abilities, nursing colleges are striving to shift from existing teaching methods, which are focused on lectures and rote learning, to diverse and scientific methods [2]. Such a shift will help improve the quality of nurse education, develop student potential, and promote well-rounded growth, so that students can gain professional competence. It is, therefore, imperative to identify educational methods that can effectively help students develop abilities in clinical reasoning and identifying and solving patient problems.

Problem-based learning (PBL) is a teaching and learning method that uses real-life situations to allow students to acquire the knowledge, skills, and attitudes needed to identify patient problems and develop the necessary solutions. Previous studies have identified PBL as an appropriate learning method in nurse education [2,3,4]. A PBL environment helps students learn independently and describe their thoughts comfortably, which can boost their confidence and self-esteem [3]. PBL fosters clinical reasoning by increasing the students’ self-efficacy and by enabling them to solve clinical problems, use clinical reasoning pathways, transfer skills to clinical practice, build knowledge as a team, and develop leadership skills [4].

Nursing students are required to engage in theoretical education and learning, as well as practice in clinical settings, to acquire extensive curricular knowledge. After entering the workforce, they also need to commit to lifelong learning, because they must adapt to dynamic changes in clinical settings. Within this context, nursing students adopt the self-directed learning (SDL) method and study proactively. SDL is a process through which learners identify their desire to learn, set their own learning goals, secure the resources necessary for learning, implement appropriate learning strategies, and assess their learning outcomes [5]. One important factor for nurses is the ability to acquire and learn professional skills independently [6]. Lifelong learning is related to the success of nursing students and the professional performance of nurses. A systematic review confirmed the importance of SDL as a learning method [7]. Another systematic review confirmed the effect of SDL on health professionals’ education, reporting its effectiveness in the domain of knowledge and skills [8]. Increased SDL can be effective in instilling and reinforcing professional nursing values in nursing students [9]. Previous studies have highlighted that self-directed learning ability (SDLA) is positively correlated with learning motivation and learning attitude [10,11] as well as with critical thinking, problem-solving [12], and academic performance [13]. In addition, access to various educational materials through digital technology has recently provided a basis for self-learning for nursing students [14].

Despite the above-mentioned findings, existing studies have had a rather fragmented focus on PBL and SDL methods [1,2,3,5,6,10,13]. It is, therefore, necessary to compare PBL and SDL directly to elucidate their influence on nursing students’ academic performance, which refers to the evaluation of learners’ acquisition of information or skills from specific classes [15]. In general, existing studies on nursing students in the university setting have shown that academic performance tends to be higher when learning motivation [16], SDLA [6,10], self-efficacy [2,6], and learning confidence [17] are high. Thus, this study assessed the effects of PBL and SDL methods in an adult nursing care curriculum for university nursing students on learning motivation, SDLA, self-efficacy, learning confidence, learning satisfaction, and academic performance.

PBL and SDL methods may differ according to topics covered, learning context, and educational objectives, but they usually involve certain established steps. To achieve educational goals through PBL, the tutor sets learning goals and forms small groups. The groups then receive goal-based case scenarios, which they must find their own ways to resolve. Subsequently, each group presents the results of their case-based problem-solving and receives feedback from their tutor and peers, followed by a discussion [18,19,20].

To achieve educational goals through SDL, the tutor sets the learning goals. Learning can be undertaken in groups, but is mainly carried out individually. The tutor provides learning topics and materials (references, online sources, video lectures, etc.), and the students acquire knowledge of the concerned topic either according to the strategy suggested by the tutor or their individual plans. Ultimately, students present their learning outcomes and receive feedback from the tutor [19,21,22].

## 2. Materials and Methods

### 2.1. Design

This quasi-experimental study employed a pre-post non-equivalent control group design.

### 2.2. Setting and Participants

This study was conducted at Semyung University, located in Chungcheongbuk-do, South Korea, between April and June 2019. Convenience sampling was used, and students were recruited through posters placed on the nursing department’s bulletin board in March. Of the 110 third-year nursing students who participated, 54 were in Class A (with a total of 84 students), and 56 were in Class B (with a total of 78 students).

Based on an independent t-test, each group required at least 51 participants, assuming a two-sided significance level (α) of 0.05, power (1−β) of 0.80, and an effect size of 0.5 [23]. Thus, the sample size of 110 was adequate.

### 2.3. Procedure

Class A was assigned to the PBL group and Class B to the SDL group. Both groups then completed a written pre-test in early April. For eight weeks, Class A was exposed to PBL-related course content while Class B was exposed to SDL-related course content. Subsequently, a post-test was completed in mid-June.

### 2.4. Preparation of PBL and SDL Materials

**Figure 1:**
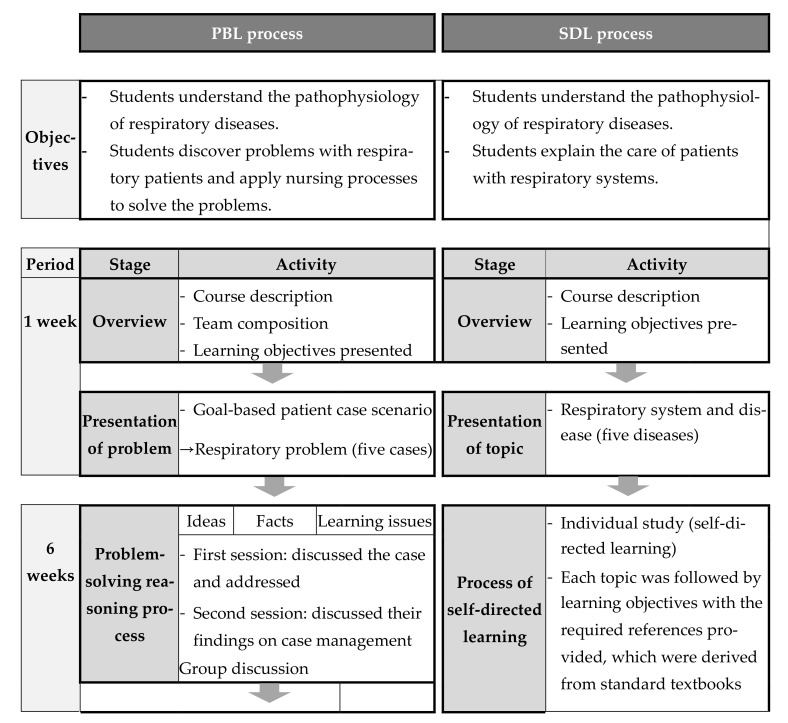

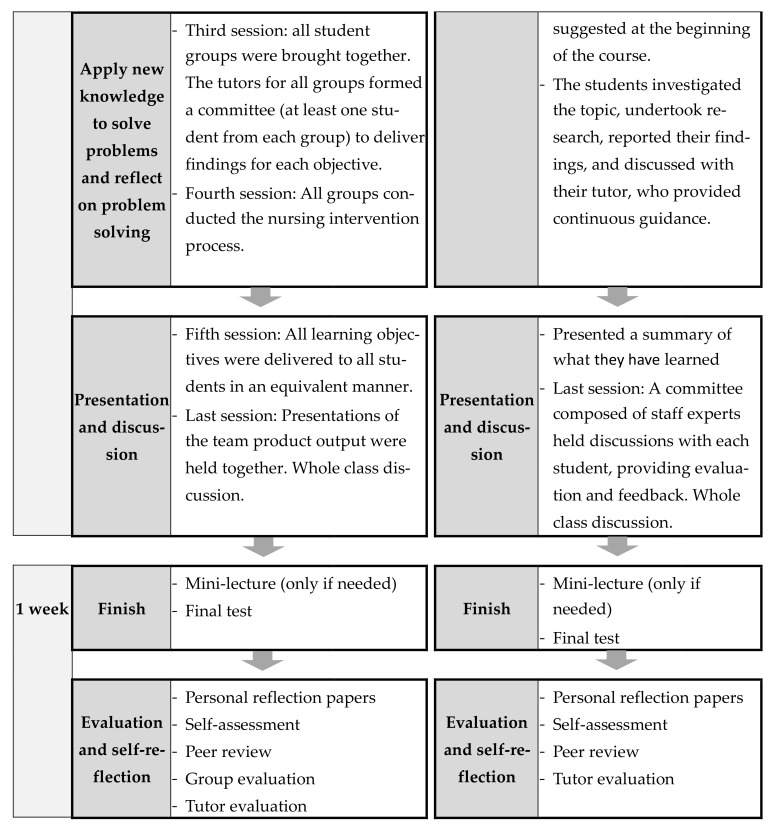
Education process of the PBL (Problem-based learning) and SDL (self-directed learning) groups.

The learning objectives and content of the adult health nursing course were designed according to Bloom’s taxonomy. Teaching tools and strategies were selected for each objective. The PBL- and SDL-specific objectives and content are presented in Figure 1.

### 2.5. Intervention

The PBL process, organized by a tutor, centers on each team solving problems based on patient scenarios on a specific topic. The objective of the PBL process is for students to solve patient case problems, apply nursing processes, and learn relevant theories. Students were divided into groups of no more than 10 each.

SDL methods are the result of self-designed or group study, and focus on self-learning of a given topic. The objective of the SDL process is for students to learn theories on a given topic.

Effective training on the application of PBL and SDL requires trained tutors or experts. In this case, the tutors selected for both PBL and SDL were staff nursing experts from different departments. The three tutors received training from the nursing education department through workshops on successful PBL and SDL that also described the role of the tutor. The PBL and SDL methods are shown in Figure 1.

### 2.6. Instruments

To assess learning motivation, Hwang’s 27-question instrument [16], which is a revised version of Keller’s 34-question survey [24], was used. Responses to items on this instrument are provided on a five-point Likert scale. Higher scores indicate higher learning motivation. As assessed through Cronbach’s alpha, the internal consistency of Keller’s instrument is 0.96 [24], and that of Hwang’s version is 0.90 [16]. In this study, Cronbach’s alpha was 0.86.

The SDLA scale, developed for university students by Lee et al. [25] and comprising 45 questions, was used in this study. Responses to items are provided on a five-point Likert scale. Higher scores indicate higher SDLA. As assessed through Cronbach’s alpha, the internal reliability of the instrument has been found to be 0.93 [25]. In this study, Cronbach’s alpha was 0.91.

Kim and Park’s [26] academic self-efficacy instrument consisting of 23 questions was used to measure participants’ self-efficacy. Responses are provided on a five-point Likert scale. Higher scores indicate greater self-efficacy. As assessed through Cronbach’s alpha, the internal reliability of the instrument has been found to be 0.84 [26]. In this study, Cronbach’s alpha was 0.79.

To measure learning confidence, an eight-item instrument developed by Hur et al. [27] was used. The instrument is based on a translation of the Self-Confidence in Learning Using Simulations Scale by Jeffries and Rizzolo [27]. It measures students’ confidence in how much their knowledge has grown over the course of taking a class. Responses are provided on a five-point Likert scale, where higher scores indicate greater learning confidence. As assessed through Cronbach’s alpha, the internal reliability of Hur et al.’s instrument has been found to be 0.70 [27]. In this study, Cronbach’s alpha was 0.72.

To assess learning satisfaction, an instrument developed by Hur et al. [27], which is a translated version of Jeffries and Rizzolo’s [28] Satisfaction in Learning Using Simulations Scale, was used. The instrument comprises five questions that measure the level of student satisfaction after a class. Items are rated on a five-point Likert scale. Higher scores indicate higher learning satisfaction. As assessed through Cronbach’s alpha, the internal reliability of the instrument developed by Hur et al. has been found to be 0.71 [27]. In this study, Cronbach’s alpha was 0.83.

Academic performance is an evaluation of the learner’s acquisition of information or skills from specific classes [15]. In this study, academic performance was evaluated using a version of Rovai et al.’s [29] nine-item instrument, developed to measure university students’ degree of academic performance in the cognitive, affective, and psychomotor domains, as adapted by Bak et al. [30]. Responses are provided on a five-point Likert scale. Higher scores indicate greater academic performance. As assessed through Cronbach’s alpha, the internal reliability of Rovai et al.’s instrument is 0.79 [29], and that of Bak et al.’s version is 0.90 [30]. In this study, Cronbach’s alpha was 0.88.

A final questionnaire was developed to evaluate the extent of students’ knowledge of the respiratory system, which is a part of the adult nursing care curriculum. Thirty questions were developed by an experienced panel of nurses and nursing professors. A correct answer was awarded one point, and a wrong answer was awarded zero points. The total possible score ranged from 0 to 30.

### 2.7. Ethical Considerations

Data were collected after the Institutional Review Board of “S” University approved the study protocol (SMU-2018-03-001-01). The employed instruments were approved for use by their respective authors. Research assistants explained the study objectives, methods, and the fact that there was no penalty for withdrawing from the study to the volunteers, who participated after providing written informed consent. Participants received small gifts for their participation. The PBL and SDL methods were cross-applied to the experimental and control groups after the post-test.

### 2.8. Data Analysis

Data were analyzed using SPSS 21.0 (IBM Corp., Armonk, NY, USA). To analyze participants’ general characteristics, we computed frequencies, percentages, means, and standard deviations. Chi-square, Fisher’s exact, and t-tests were used to test for homogeneity between the PBL and SDL groups with regard to general characteristics and pre-intervention dependent variables. After each learning method was applied to the PBL and SDL groups, the Kolmogorov–Smirnov normality test was performed and its conditions were satisfied. Subsequently, a paired t-test and an independent t-test were performed to examine differences in learning motivation, SDLA, self-efficacy, learning confidence, learning satisfaction, and academic performance, before and after the intervention. Thirty questions representing SDL and PBL were included in the final examination, and comparisons were made through an independent t-test and a chi-square test. For each tool, student scores (maximum of 30) were categorized into high (≥24), moderate (18–23), and low (<17). For all analyses, *p* ˂ 0.05 was considered significant.

## 3. Results

### 3.1. Participants’ General Characteristics and Homogeneity Test Results

After excluding four students—two members of the SDL group and one member of the PBL group who did not take part in the post-test, and one member of the PBL group who took a leave of absence during the semester—the data from 106 participants (52 from the PBL group, 54 from the SDL group) were analyzed. Participant characteristics are shown in Table 1. As per the homogeneity test of participants’ general characteristics, there were no significant differences between the PBL and SDL groups, thereby confirming homogeneity between the two groups.

### 3.2. Homogeneity Test of the Groups’ Pre-Intervention Dependent Variables

The t-test results showed no significant difference between the PBL and SDL groups in any of the pre-intervention dependent variables, thus confirming between-group homogeneity (Table 2).

### 3.3. Differences between the PBL and SDL Groups in Intervention Outcomes

In the PBL group, there were differences between pre-test and post-test scores for learning motivation (*t* = −2.262, *p* = 0.031), SDLA (*t* = −1.066, *p* = 0.045), self-efficacy (*t* = −2.177, *p* = 0.037), and satisfaction in learning (*t* = −1.938, *p* = 0.042). There were differences in the SDL group’s pre-test and post-test scores for SDLA (*t* = −1.360, *p* = 0.049) and self-efficacy (*t* = −1.240, *p* = 0.045). A comparison of the PBL and SDL groups’ pre-test and post-test scores showed statistically significant differences in learning motivation (*t* = 2.265, *p* = 0.027), SDLA (*t* = 1.506, *p* = 0.048) and satisfaction in learning (*t* = 1.580, *p* = 0.037; Table 3).

### 3.4. Difference between PBL and SDL Scores in the Final Test

The PBL group’s average score was 22.85 ± 3.74, while that of the SDL group was 20.94 ± 3.39. The difference between the two groups was statistically significant (*t* = 2.746, *p* = 0.007). Based on categorization of the scores according to high, moderate, and low, the PBL group had a higher distribution of participants scoring “high” than did the SDL group, and a lower distribution of participants scoring “low”; the difference was statistically significant (χ^2^ = 2.146, *p* = 0.046; Table 4).

## 4. Discussion

This study aimed to compare the effectiveness of the PBL method which is used to solve problems in patient cases, and the SDL method which is used for self-learning on a topic, in adult nursing student education. The PBL group demonstrated a significant increase in learning motivation, SDLA, self-efficacy, and satisfaction in learning. The PBL and SDL groups did not differ statistically in self-efficacy, learning confidence, and academic performance. The PBL group’s respiratory system knowledge, as demonstrated by scores on the final test, however, was significantly higher than that of the SDL group. This result warrants further review. The implication of this study is that, to maximize the effect of SDL education, it is necessary to devise an educational method that includes the PBL method as well, as it was more effective than the SDL method in nursing education.

Regarding learning motivation, the SDL group’s post-test score was much lower than that of the PBL group. Studies have reported that nurses who used the SDL method experienced learning difficulties at many stages, resulting in a lack of learning motivation [21]. Furthermore, it has been reported that learning motivation is correlated with SDL readiness and this affects learning satisfaction [31]. The present study found that the SDL group’s learning satisfaction score did not change as much as that of the PBL group. The positive impact of PBL on nursing students’ learning motivation is supported by prior studies [2,18,32].

Both the PBL and the SDL groups demonstrated an increase in post-test scores on the SDLA scale, compared to pre-test scores. SDLA is a quality that is expected of healthcare graduates [26]. Both SDL and PBL have previously been found to improve SDLA [27]. However, through small group discussions, PBL was shown to be more effective in improving SDLA [20]. This finding is consistent with the present study; our post-test SDLA score showed a greater increase in the PBL group than in the SDL group.

The term “self-directed” in SDLA may sometimes be misleading; it could imply that no help is needed in learning [5]. When the SDL readiness of a lecture-type group and the PBL group were compared, there was no difference in self-management, desire for learning, or self-control [1]. This suggests that SDLA is not an ability that can be naturally enhanced simply by adopting a different learning method; it needs strategic programs for improvement [1]. Therefore, further studies should review whether the PBL and SDL methods used in previous studies were indeed effective in improving SDLA, and identify which of the two methods is more effective. It is important to encourage students to maintain an environment that allows them to make ongoing efforts to improve their SDLA.

Regarding self-efficacy, we found a significant increase in the post-test scores of both groups. This finding is supported by previous studies with nursing students [2], medical students [20], and nurses [33]. Self-efficacy was also found to positively affect nursing students’ academic performance [2,6]. These findings indicate that both PBL and SDL improve nursing students’ self-efficacy, thereby influencing their academic performance. It is, thus, necessary to use both methods to enhance students’ self-efficacy.

Adult nursing care is a vast and challenging curriculum that demands the coverage of a wide range of topics in a brief period. The application of SDL [17] and PBL [34] in education improves nursing students’ learning confidence; however, the intervention duration in prior studies was 14 weeks, and our eight-week intervention may not have been sufficient to bolster students’ learning confidence. The post-test scores for learning confidence in our study increased for both the PBL and SDL groups, although pre-post differences were not significant.

In the PBL group, the pre-post difference in learning satisfaction scores was significant; this was not the case in the SDL group. A previous meta-analysis revealed that PBL required professors to be professionally skilled in clinical reasoning and in preparing students to navigate this method [31]. Thus, it is important to review the skills and preparedness of professors who teach PBL. Conversely, for SDL to be effective, nursing students need SDL readiness. Culture and curriculum play a vital role in ensuring SDL readiness, suggesting the need for strategies to develop appropriate learning environments that ensure SDL effectiveness [22]. Access to professionals who can model the application of content to nursing is tied to the need for discipline-specific facilitators with at least some knowledge of nursing, rather than generic facilitators of learning (including the student as a self-directed facilitator of learning).

The pre-post score difference in academic performance was also non-significant. However, in the final test, the PBL group obtained a higher average score and a higher distribution of “high” scores compared to the SDL group, demonstrating a more positive impact of PBL on academic achievement. Findings from other studies also show that PBL [32] and SDL [1] positively influence nursing students’ academic performance. The lack of significant improvement in academic performance is consistent with the findings of an earlier study on PBL among nursing students, which showed that readiness for SDL had an inverse, significant relationship with two of five exam scores. Specifically, students’ belief in their readiness for SDL did not always produce course content mastery [32]. Therefore, further strategies are needed to improve nursing students’ academic performance.

In a systematic review of effective SDL strategies in medical students, the included studies focused on setting goals and monitoring the situation and social relations, not on the SDL process and self-assessment support, thus limiting the effectiveness of SDL [35]. The systematic review also revealed a gap in research focusing on uncovering the SDL process and assessing students’ progress toward their goals [35]. It is known that SDL helps improve the learning effect of nursing students; however, the process of SDL has not yet been systematically revealed [7]. In this context, the present study fills a research gap by demonstrating that, to maximize learning in adult nursing education, it is necessary to apply the SDL method, including the PBL method, with a clearer learning process. Further research is needed to confirm the effectiveness of SDL education, including the PBL method, in adult nursing university curriculum.

Despite its strengths, this study has certain limitations. First, owing to the use of five variables to confirm the difference in the effects of PBL and SDL, the questionnaire contained a large number of items. This could have led to fatigue during the pre- and post-test, reducing the accuracy of participants’ responses. Second, this study was conducted in only one school, which limits the generalizability of the results. Finally, the eight-week intervention period may not have been sufficient to demonstrate changes in learning motivation, SDLA, self-efficacy, learning confidence, and learning satisfaction, or to improve students’ academic performance.

## 5. Conclusions

This study attempted to explore the effects of applying the SDL and PBL methods to adult nursing education. SDL is an important aspect of lifelong learning in an integration-based curriculum. However, the SDL method was less effective than the PBL method in nurse education in an adult nursing curriculum. In future studies, we will continue using well-planned PBL methods for nursing student education and make recommendations for the development and application of evidence-based SDL methods. Based on this study, a further exploration of PBL curriculum development in nursing education is suggested. We also suggest that future research should explore the development of the SDL learning process by including the PBL method.

## Figures and Tables

**Table 1:** Baseline characteristics of the PBL and SDL groups.

Characteristics	Categories	PBL (*n* = 52) *n* (%)	SDL (*n* = 54) *n* (%)	χ^2^	*p*
Gender	Women	40 (76.9)	39 (72.2)	0.333 *	0.774
	Men	12 (23.1)	15 (27.8)		
Age (years)	19–20	21 (40.4)	22 (40.7)	10.915	0.142
	21–25	31 (59.6)	32 (59.3)		
	Mean ± SD	21.09 ± 1.25	21.66 ± 2.21		
Motive for department choice	Aptitude and interest	18 (34.6)	20 (37.0)	7.458 *	0.114
	High employment rate	27 (51.9)	19 (35.2)		
	Academic record	2 (3.8)	10 (18.5)		
	On recommendation	5 (9.7)	5 (9.3)		
Satisfaction with major	Dissatisfied	6 (11.5)	8 (14.8)	4.253 *	0.119
	Average	25 (48.1)	18 (33.3)		
	Satisfied	21 (40.4)	26 (51.9)		
Average grade	2.0–2.99	8 (15.4)	10 (18.5)	12.509 *	0.114
	3.0–3.49	16 (30.7)	15 (27.8)		
	3.5–3.99	22 (42.3)	23 (42.6)		
	≥4.0	6 (11.6)	6 (11.1)		

Note. χ^2^ = chi-square; * Fisher’s exact test; PBL: problem-based learning; SDL: self-directed learning; SD: standard deviation.

**Table 2:** Homogeneity of dependent variables pre-intervention.

Variable	PBL (*n* = 52) Mean ± SD	SDL (*n* = 54) Mean ± SD	*t*	*p*
Learning motivation	79.09 ± 6.42	80.69 ± 9.67	0.777	0.440
Self-directed learning ability	141.34 ± 15.55	138.13 ± 16.99	1.036	0.304
Self-efficacy	71.78 ± 6.14	68.16 ± 8.00	2.033	0.056
Self-confidence in learning	24.25 ± 2.48	23.66 ± 3.76	0.746	0.459
Satisfaction in learning	17.59 ± 2.82	17.25 ± 2.53	0.415	0.609
Academic performance	28.44 ± 3.57	26.88 ± 5.93	1.276	0.207

Note. SD: standard deviation; PBL: problem-based learning; SDL: self-directed learning.

**Table 3:** Pre-post comparison of study variables.

Variable	Group	Pre-Test Mean ± SD	Post-Test Mean ± SD	*t* * (*p)*	Difference Mean ± SD	*t* **	*p*
Learning motivation	PBL (*n* = 52)	79.09 ± 6.42	84.59 ± 8.53	−2.262 (0.031)	5.50 ± 8.75	2.265	0.027
	SDL (*n* = 54)	80.69 ± 9.67	80.81 ± 9.36	1.042 (0.305)	0.11 ± 10.18		
Self-directed learning ability	PBL (*n* = 52)	141.34 ± 15.55	149.16 ± 12.39	−1.066 (0.045)	7.81 ± 14.92	1.506	0.048
	SDL (*n* = 54)	138.13 ± 16.99	142.0 ± 18.40	−1.360 (0.049)	3.88 ± 15.68		
Self-efficacy	PBL (*n* = 52)	71.78 ± 6.14	76.19 ± 5.26	−2.177 (0.037)	4.41 ± 6.25	1.318	0.054
	SDL (*n* = 54)	68.16 ± 8.00	72.44 ± 7.31	−1.240 (0.045)	4.28 ± 6.64		
Self-confidence in learning	PBL (*n* = 52)	24.25 ± 2.48	27.22 ± 3.01	−1.453 (0.156)	2.97 ± 3.77	1.601	0.114
	SDL (*n* = 54)	23.66 ± 3.76	24.97 ± 3.54	0.869 (0.392)	1.31 ± 4.48		
Satisfaction in learning	PBL (*n* = 52)	17.59 ± 2.82	23.06 ± 2.95	−1.938 (0.042)	5.47 ± 4.29	1.580	0.037
	SDL (*n* = 54)	17.25 ± 2.53	18.38 ± 3.42	0.188 (0.852)	1.13 ± 3.77		
Academic performance	PBL (*n* = 52)	28.44 ± 3.57	31.56 ± 3.82	−1.327 (0.194)	3.13 ± 4.80	0.371	0.712
	SDL (*n* = 54)	26.88 ± 5.93	29.53 ± 4.72	−0.700 (0.489)	2.66 ± 5.30		

Note. SD: standard deviation; PBL: problem-based learning; SDL: self-directed learning; * paired *t*-test; ** independent *t*-test.

**Table 4:** Differences in the PBL and SDL groups’ final test scores.

Final Test	Group		*t* or χ^2^ (*p*)
	PBL (*n* = 52)	SDL (*n* = 54)	
Total score (0–30), mean ± SD	22.85 ± 3.74	20.94 ± 3.39	2.746 (0.007)
High, *n* (%)	22 (42.3)	12 (22.2)	2.146 (0.046)
Moderate, *n* (%)	23 (44.2)	30 (55.6)	
Low, *n* (%)	7 (13.5)	12 (22.2)	

Note. χ^2^ = chi-square; PBL: problem-based learning; SDL: self-directed learning; SD: standard deviation.

## Data Availability

Not applicable.
